# Dear Pandemic: A topic modeling analysis of COVID-19 information needs among readers of an online science communication campaign

**DOI:** 10.1371/journal.pone.0281773

**Published:** 2023-03-30

**Authors:** Aleksandra M. Golos, Sharath Chandra Guntuku, Rachael Piltch-Loeb, Lindsey J. Leininger, Amanda M. Simanek, Aparna Kumar, Sandra S. Albrecht, Jennifer Beam Dowd, Malia Jones, Alison M. Buttenheim

**Affiliations:** 1 Department of Family and Community Health, School of Nursing, University of Pennsylvania, Philadelphia, PA, United States of America; 2 Department of Computer and Information Science, School of Engineering and Applied Science, University of Pennsylvania, Philadelphia, PA, United States of America; 3 Leonard Davis Institute of Health Economics, University of Pennsylvania, Philadelphia, PA, United States of America; 4 Department of Biostatistics, Harvard T.H. Chan School of Public Health, Harvard University, Boston, MA, United States of America; 5 Emergency Preparedness Research Evaluation and Practice Program, Harvard T.H. Chan School of Public Health, Harvard University, Boston, MA, United States of America; 6 Tuck School of Business, Dartmouth College, Hanover, NH, United States of America; 7 Joseph J. Zilber School of Public Health, University of Wisconsin-Milwaukee, Milwaukee, WI, United States of America; 8 College of Nursing, Thomas Jefferson University, Philadelphia, PA, United States of America; 9 Department of Epidemiology, Mailman School of Public Health, Columbia University, New York, NY, United States of America; 10 Leverhulme Centre for Demographic Science, University of Oxford, Oxford, United Kingdom; 11 Department of Sociology, University of Oxford, Oxford, United Kingdom; 12 Nuffield College, University of Oxford, Oxford, United Kingdom; 13 Applied Population Laboratory, Department of Community and Environmental Sociology, College of Agricultural and Life Sciences, University of Wisconsin-Madison, Madison, WI, United States of America; 14 Center for Health Incentives and Behavioral Economics, University of Pennsylvania, Philadelphia, PA, United States of America; Bogazici University, TURKEY

## Abstract

**Background:**

The COVID-19 pandemic was accompanied by an “infodemic”–an overwhelming excess of accurate, inaccurate, and uncertain information. The social media-based science communication campaign Dear Pandemic was established to address the COVID-19 infodemic, in part by soliciting submissions from readers to an online question box. Our study characterized the information needs of Dear Pandemic’s readers by identifying themes and longitudinal trends among question box submissions.

**Methods:**

We conducted a retrospective analysis of questions submitted from August 24, 2020, to August 24, 2021. We used Latent Dirichlet Allocation topic modeling to identify 25 topics among the submissions, then used thematic analysis to interpret the topics based on their top words and submissions. We used t-Distributed Stochastic Neighbor Embedding to visualize the relationship between topics, and we used generalized additive models to describe trends in topic prevalence over time.

**Results:**

We analyzed 3839 submissions, 90% from United States-based readers. We classified the 25 topics into 6 overarching themes: ‘Scientific and Medical Basis of COVID-19,’ ‘COVID-19 Vaccine,’ ‘COVID-19 Mitigation Strategies,’ ‘Society and Institutions,’ ‘Family and Personal Relationships,’ and ‘Navigating the COVID-19 Infodemic.’ Trends in topics about viral variants, vaccination, COVID-19 mitigation strategies, and children aligned with the news cycle and reflected the anticipation of future events. Over time, vaccine-related submissions became increasingly related to those surrounding social interaction.

**Conclusions:**

Question box submissions represented distinct themes that varied in prominence over time. Dear Pandemic’s readers sought information that would not only clarify novel scientific concepts, but would also be timely and practical to their personal lives. Our question box format and topic modeling approach offers science communicators a robust methodology for tracking, understanding, and responding to the information needs of online audiences.

## Introduction

The COVID-19 pandemic spurred a global communication crisis that the World Health Organization (WHO) named an “infodemic.” Defined as a problem of too much information–including accurate information, rapidly evolving guidance, and misinformation–the COVID-19 infodemic eroded trust in science and undermined compliance with public health measures [1]. As seeking information became more complicated, it also became more consequential: exposure to credible COVID-19 information may have increased worry about disease susceptibility and severity, prompting people to engage in COVID-19 mitigation strategies [2]. Conversely, exposure to misinformation may have led people to heuristic processing and information avoidance, entrenching detrimental health beliefs [3]. The WHO developed an infodemic management framework to help science communicators address this crisis; it recommends conducting information surveillance, translating expert knowledge into accessible language, bolstering scientific literacy, and fact-checking information [4, 5]. These efforts should happen through two-way, rather than one-way, engagement with the public [6].

The science communication campaign Dear Pandemic was established in March 2020 to address the COVID-19 infodemic via two-way engagement on Instagram, Facebook, and Twitter. Run by an interdisciplinary team of clinicians and researchers, Dear Pandemic is responsive to readers’ information needs, translating emerging news and science into actionable guidance. To more effectively engage with readers, Dear Pandemic opened an online “question box” in August 2020. Readers submit questions via a simple online webform, and Dear Pandemic contributors use the questions to inform upcoming content. This approach was highly successful; by late 2021, the campaign had 180,000 regular readers from over 60 countries, and its content reached over 1 million unique monthly views.

Dear Pandemic’s question box presents a unique opportunity to understand the information needs of an engaged online audience during the COVID-19 pandemic. Though other studies have analyzed online COVID-19 discourse, most relied on broad sets of keywords to identify relevant content or did not discern between different types of discourse [7–10]. Among the studies that focused on information-seeking, Mangono et al. and Chan & Chua used Google search volume as a proxy for information needs during the onset of the pandemic [11, 12]. Kim & Oh used a dataset of COVID-19 related posts on the South Korean question-and-answer platform Naver Knowledge iN, allowing for a more direct analysis of information needs [13]. However, their dataset was limited to posts from February 1 to October 31, 2020, and questions were answered by the lay public rather than by experts with specialized, fact-checked knowledge. Little is therefore known about discourse written for the purpose of seeking credible information about COVID-19. Our study makes significant contributions to the literature on COVID-19 information needs by analyzing data through August 2021 (covering the development and rollout of the COVID-19 vaccine) and focusing on audiences who sought information from trusted science communicators.

Many of the aforementioned studies have used Latent Dirichlet Allocation (LDA) topic modeling to analyze online COVID-19 discourse. LDA is a quantitative method that groups words with high probabilities of co-occurrence into topics. Each text input to the model is described by a probability distribution of topics into which it falls, and each topic is described by a distribution of highly-associated words [14]. LDA has often been used to analyze large-scale datasets (e.g., tweets) [7, 9, 10], but it is also well-suited for short-text data and small corpora [15], making it appropriate for our dataset. Murakami et al. described the advantages of LDA topic modeling in the exploration of a specialized corpus, highlighting its utility in identifying different types of content, differentiating multiple senses of words, and examining chronological changes [16]. A further advantage of LDA is that it allows for researcher input in naming, interpreting, and categorizing topics, providing a deeper understanding of the results. We harnessed this by using established qualitative methods of thematic analysis to identify overarching patterns, or “themes,” among the topics [17].

In this study, we use LDA and thematic analysis to 1) identify topics among reader-submitted questions to Dear Pandemic’s question box; 2) visualize the association between topics; and 3) describe trends in topic prevalence over time. By characterizing the information needs of Dear Pandemic’s audience, we not only provide other science communicators with insights about information-seeking during COVID-19, but also illustrate how our question box format and topic modeling approach can be useful for infodemic management.

## Materials and methods

### Data collection and pre-processing

S1 Fig displays the research process, from data collection to visualization. Our data consisted of submissions to Dear Pandemic’s question box from August 24, 2020, to August 24, 2021. We chose a year-long period in order to cover a wide range of the rapidly shifting COVID-19 information landscape, while also limiting the inclusion of extraneous questions that were submitted in late 2021 as Dear Pandemic broadened its scope to other health and science topics. To assemble our corpus, we retrospectively collected all 3947 questions that readers submitted via webform during the analysis period. The webform also included a field for readers to report their geographic location, which we categorized by US state and/or country. After removing 108 duplicate or non-English-language questions, we obtained a final corpus of 3839 submissions.

We pre-processed the text of each submission by converting it to lowercase and removing numbers, special characters, stop words (i.e., common words with little meaning), and URLs. Finally, we tokenized (i.e., split the submission text into individual words) and lemmatized (i.e., converted words into their root form) the text using the Python library spaCy [18]. The mean (SD) submission length was 66.7 (61.7) words originally and 31.5 (29.7) tokens after pre-processing.

### Topic modeling

To analyze submissions, we used an LDA topic modeling algorithm provided by the Differential Language Analysis ToolKit (DLATK) Mallet interface [19, 20]. We adjusted one parameter (alpha = 0.3) to favor fewer topics per document (i.e., submission), reflecting the fact that our documents were relatively short in length (compared to documents such as book chapters or news articles) and were thus likely to contain fewer topics. All other LDA parameters were kept at their default.

The number of topics to generate, *k*, must be manually pre-specified in LDA models. This parameter can be optimized by comparing the results of models run with different values of *k*, using quantitative topic coherence metrics and qualitative measures of interpretability. We estimated a series of models with 3 < *k* < 50 topics and calculated coherence using the C_v_ method [21]. We also reviewed the topics generated in each iteration for semantic validity (whether topic meanings could be clearly discerned by their associated words) and granularity (whether topics were broad or specific). While the 9-topic model had the highest C_v_ score (S2 Fig), we judged the topics to be insufficiently granular. We reached the consensus that the 25-topic model was optimal based on its semantic validity, granularity, and C_v_ score.

After the model was finalized, 5 of the authors with expertise in qualitative research conducted a thematic analysis of the topics, following the process described by Braun & Clarke [17]. To integrate LDA and thematic analysis, we followed the best practice of identifying the words and documents that were most highly associated with each topic [22]. We calculated log-likelihood word frequencies to identify the top 50 words in each topic, and we used submission-level probability distributions to identify the top 10 submissions in each topic. The 5 authors independently familiarized themselves with the data; generated names and descriptions for each of the 25 topics using the top words and submissions; and grouped the 25 topics into candidate themes based on common features and meanings. The authors identified 11 candidate themes, which were compared, mapped, and consolidated until 6 final themes were generated. The names and interpretations of the topics and themes were refined until a consensus among all authors was reached.

Finally, we visualized the submissions using a t-distributed stochastic neighbor embedding (t-SNE) algorithm, which represented each submission’s 25-dimensional topic probability distribution in a 2-dimensional space. To examine trends over time, we computed each topic’s average probability by day and fit generalized additive models to the data. We visualized the data and conducted trend analyses using R version 4.0.3. Our study was determined to not meet the definition of human subjects research by the University of Pennsylvania Institutional Review Board, waiving the requirement for informed consent.

## Results

### Characteristics of submissions to the Dear Pandemic question box

The topic model included 3839 question box submissions. The number of daily submissions ranged from 1 to 32, and the most active month was March 2021, with 592 submissions (S3 Fig). 90.0% of submissions came from US-based readers; the 5 states with the most submissions were Wisconsin (417), Texas (333), Pennsylvania (265), New York (244), and California (234) (S1 Data). Other countries with 5 or more submissions were Canada (96), the United Kingdom (67), Australia (12), Mexico (6), South Africa (5), India (5), and Germany (5). These frequencies generally map to the location of Dear Pandemic readers on social media. As Dear Pandemic periodically promoted the question box during media interviews and events, submission volume and geographic distribution may not have been solely organic.

### Themes and topics identified among submissions to the Dear Pandemic question box

Table 1 provides an overview of the 25 topics, including each topic’s 20 most frequently associated words, an example submission, the number of submissions for which each topic was the highest-probability topic, and each topic’s mean probability. Topic probabilities were low on average, highly variable, and highly right-skewed; relatively few submissions had high probabilities of being represented by a single topic (S2 Data). The following results describe our qualitative interpretation of the topics and themes.

**Table 1:** Overview of themes and topics among submissions to the Dear Pandemic question box.

Topic name	Most frequently associated words	Example submission	Number (%) of submissions^a^	Mean (SD) topic probability^b^
**Theme 1: Scientific and medical basis of COVID-19**				
Immunology	immune, antibody, disease, system, vaccination, response, cell, spike, blood, treatment, protein, autoimmune, body, mrna, produce, medication, patient, treat, specifically, develop	Can the Pfizer vaccine alter or weaken my existing cross-reactive antibodies / immune system?	209 (5.34)	4.01 (6.70)
Disease process	covid, long, term, infection, symptom, mild, severe, concern, case, effect, prevent, issue, health, hear, may, heart, damage, disease, serious, illness	How do you know if you have heart damage post covid?	176 (4.58)	4.40 (5.33)
Immunity and transmission	virus, people, immunity, still, person, vaccinate, infection, infect, someone, could, even, spread, transmit, other, herd, understand, contract, asymptomatic, viral, likely	Why is there little to no talk about natural immunity from exposure or infection in terms of reaching herd-immunity?	173 (4.51)	4.65 (5.23)
Viral variants	variant, new, delta, virus, concern, transmission, spread, come, strain, sar, evidence, mean, possible, base, show, cov, protect, coronavirus, uk, infect	Is the delta variant more severe for children than the other variants?	142 (3.70)	3.41 (5.80)
Other medical conditions	get, vaccine, covid, flu, tell, sick, able, wait, shoot, try, already, mom, never, type, yet, thing, eligible, chance, turn, stop	Should we get the flu shot earlier this year? We usually get ours in October, but I’m nervous about the double whammy of seasonal flu and Covid in schools.	89 (2.32)	3.70 (4.18)
**Theme 2: COVID-19 vaccine**				
Dosage and timing	dose, get, first, second, shot, pfizer, week, moderna, receive, one, two, nd, booster, shoot, wait, month, arm, effective, protection, schedule	The vaccine is supposed to be effective for six months. Is that six months from the first shot, the second shot, or from the two weeks after the second shot?	281 (7.32)	5.14 (7.60)
Development and rollout	vaccine, trial, study, available, pfizer, efficacy, mrna, johnson, approve, moderna, safety, receive, datum, pregnant, effective, currently, approval, fda, develop, woman	Dear Pandemic, what is the difference between emergency authorization for a vaccine, and "regular" authorization?	212 (5.52)	4.68 (6.27)
Behavior surrounding vaccination	vaccinated, vaccinate, fully, people, unvaccinated, indoor, child, household, cdc, person, guideline, adult, guidance, visit, without, different, husband, unmasked, grandparent, change	Can fully vaccinated people socialize with other fully vaccinated people? With or without masks? How about eating in a restaurant?	197 (5.13)	4.64 (5.72)
Safety and side effects	vaccine, effect, side, reaction, take, report, cause, receive, response, woman, allergy, concern, might, allergic, affect, bad, experience, body, pain, immune	Are the vaccine doses for women smaller than for men? I know more women that have adverse reactions to the vaccines than men.	182 (4.74)	3.93 (6.30)
**Theme 3: COVID-19 mitigation strategies**				
Socializing safely	mask, wear, distance, outside, kid, safe, outdoor, indoor, outdoors, social, play, distancing, even, foot, other, etc, summer, friend, apart, everyone	I take brisk walks outside with a friend. We stay approximately six feet apart but do not wear masks. How safe is this?	232 (6.04)	4.60 (6.60)
Testing and isolation	test, covid, positive, day, quarantine, symptom, negative, testing, expose, antibody, result, week, exposure, contact, someone, pcr, month, rapid, tell, recently	I am vaccinated and recently tested positive. My only symptom is no taste/smell. Do I still have to quarantine for 10 days?	229 (5.97)	4.57 (6.41)
Ventilation	air, office, room, open, work, window, hour, can, not, indoor, small, someone, space, door, building, share, close, house, ventilation, line	I live in an apartment building. Is it true that I can catch covid from air that is in other apartments around me that comes through my air ducts? Should I install air filters?	128 (3.33)	3.22 (4.90)
Sanitation and hygiene	hand, need, grocery, eat, surface, use, food, store, cold, touch, wash, really, every, transmission, leave, place, avoid, fomite, restaurant, thing	What’s the latest on fomite transmission? Do we need to wash our hands when we get the mail?	117 (3.05)	2.98 (5.61)
Masks	mask, wear, well, protect, protection, face, public, good, double, recommend, course, cloth, effective, recommendation, require, clear, way, kn, provide, talk	Which masks provide the most protection for kids in school? (Filter and cloth, medical or KN95).	91 (2.37)	3.01 (4.88)
**Theme 4: Society and institutions**				
Education	school, student, kid, teacher, person, back, classroom, require, decision, class, district, open, return, reopen, send, full, learn, fall, guidance, elementary	What mitigating steps can school leaders / individual teachers put in place to reduce spread when schools reopen to all pupils?	163 (4.25)	3.54 (5.23)
Healthcare and policy	health, community, care, worker, county, state, public, medical, follow, guidance, rate, area, healthcare, local, group, right, patient, hospital, continue, number	Are health care providers still getting covid? Is their PPE really working? Do all health care providers have sufficient PPE now?	87 (2.27)	2.91 (4.09)
Social participation	travel, vaccination, state, month, post, early, return, last, country, summer, may, event, end, soon, plan, late, expect, safely, fall, look	When will the US / Canadian border be open and allow for travel without quarantine restrictions?	73 (1.90)	3.07 (4.30)
**Theme 5: Family and personal relationships**				
Interacting with family	family, would, visit, safe, see, home, stay, parent, we, member, quarantine, plan, precaution, live, travel, together, come, house, husband, possible	Can I visit my family for Christmas if we’ve all been taking COVID seriously?	205 (5.34)	4.48 (5.50)
Children and parenting	kid, child, year, old, young, adult, age, parent, son, healthy, small, able, daughter, great, start, group, fall, toddler, eligible, yo	What are parents of young children supposed to do with the new CDC guidance about masklessness?	175 (4.56)	4.36 (5.29)
Personal relationships	home, go, we, daughter, want, husband, stay, also, friend, day, back, since, son, come, live, two, start, around, couple, march	Can I let my neighbors (children) who go to in-person school and play with friends pet my dog?	87 (2.27)	3.69 (3.92)
**Theme 6: Navigating the COVID-19 infodemic**				
Verifying information	say, see, article, please, make, post, study, read, something, find, explain, claim, dr, hear, true, information, link, ask, news, cdc	Can you comment on the science behind the new claims from Geert Vanden Bossche?	154 (4.01)	4.26 (5.89)
Feedback	thank, question, love, much, post, answer, wonder, think, appreciate, good, pandemic, information, share, info, girl, thought, help, address, hi, provide	This is just a thank you for your answers and you really helpful job! Thank you!	144 (3.75)	4.22 (5.15)
Data and statistics	case, death, rate, number, datum, seem, infection, spread, report, less, percentage, low, die, read, increase, age, example, hospital, information, among	Should we paying attention to case count per 100,000 or positivity rates to gauge community spread? What does it mean when positivity is low but case count high?	137 (3.57)	3.92 (4.89)
Sense-making	people, like, feel, seem, many, thing, even, normal, really, want, keep, life, right, pandemic, lot, maybe, less, happen, social, little	Honest question—how do you deal with vocal vaccine deniers? I don’t want them polluting the vast majority of the population into not vaccinating, and it is honestly scary. Thanks from the UK.	103 (2.68)	4.37 (3.91)
Risk assessment	risk, high, low, seem, make, level, reduce, transmission, good, understand, factor, consider, tell, try, study, way, exposure, everyone, without, whether, follow, big, kind, show, regard	I was wondering if you could create a chart where one side lists the risks associated with the vaccine and the other side lists the risks associated with the virus.	53 (1.38)	3.66 (3.66)

^a^ Determined based on the number of submissions for which each topic was the highest-probability topic in the distribution.

^b^ Calculated using the full topic probability distribution for each submission.

### Theme 1: Scientific and medical basis of COVID-19

This theme contained 5 topics related to scientific and medical aspects of the pandemic. ‘Immunology’ covered questions about the immune system, the SARS-CoV-2 virus, and vaccines. ‘Disease Process’ was related to the symptoms and sequelae of COVID-19, distinguishing between “mild,” “severe,” and “long” COVID. ‘Immunity and Transmission’ reflected uncertainties about viral transmission and infection- or vaccine-induced immunity. ‘Viral Variants’ mainly referenced the Delta variant, though the “UK” (i.e., Alpha) variant was also mentioned. Finally, submissions in ‘Other Medical Conditions’ raised concerns about comorbid conditions and infection or vaccination. “Flu” was the most frequently mentioned disease or condition, though others among the top 50 included “pneumonia,” “asthma,” “obese,” “arthritis,” and “diabetes.” Other frequent words reflected uncertainty about timing (e.g., “able,” “wait,” “eligible”).

### Theme 2: COVID-19 vaccine

This theme contained 4 topics specifically related to the COVID-19 vaccine. ‘Dosage and Timing’ captured general questions about the vaccination schedule, as well as words that provided context about readers’ vaccination status and time since vaccination (e.g., “first,” “Moderna,” “month”). Submissions in ‘Development and Rollout’ were related to the vaccine’s research and development process, its rollout, and regulatory agencies. Words indicating objective metrics (e.g., “datum [data],” “efficacy,” “effective”) were frequent. ‘Behavior Surrounding Vaccination’ captured considerations about socializing given vaccination status and official guidelines. Finally, ‘Safety and Side Effects’ reflected fears about adverse effects (e.g., “report,” “cause,” “concern”). Safety concerns were sometimes gender-specific; the word “woman” was frequently mentioned, and “fertility,” “menstrual,” and “cycle” were also among the top 50 words.

### Theme 3: COVID-19 mitigation strategies

This theme contained 5 topics related to COVID-19 mitigation strategies aside from vaccination. ‘Socializing Safely’ contained situation-specific questions about mitigating personal risk via non-pharmaceutical interventions (e.g., “mask,” “outdoor,” “distancing”). ‘Testing and Isolation’ was related to best practices following exposure or infection. Though “asymptomatic” was among the top 50 words associated with this topic, no words were explicitly related to vaccination. ‘Ventilation’ was specific to mitigating airborne transmission, including questions about airflow in homes, schools, and workplaces. ‘Sanitation and Hygiene’ was specific to mitigating surface transmission. Activities surrounding food (e.g., “grocery,” “food,” “restaurant”) were frequently referenced. Finally, ‘Masks’ reflected the desire to optimize face mask usage (e.g., “protect,” “double,” “effective”). References to mask “recommend[ations]” or “require[ments]” were also common.

### Theme 4: Society and institutions

This theme contained 3 topics related to social institutions. The most frequent words in ‘Education’ (e.g., “decision,” “reopen,” “guidance”) reflected uncertainty surrounding the return to in-person schooling, especially for “elementary” school-aged children. ‘Healthcare and Policy’ was a broad topic covering the healthcare system, public policy, official COVID-19 guidelines, and occupations. Many submissions referenced specific locations. Finally, ‘Social Participation’ captured participation in public life, most notably “travel.” Guidelines or requirements (e.g., “vaccination”), locations (e.g., “state,” “country”), and timing or anticipation (e.g., “month,” “summer,” “plan”) were frequently referenced.

### Theme 5: Family and personal relationships

This theme contained 3 topics that mostly captured submissions seeking situation-specific advice. ‘Interacting with Family’ covered interactions within and between households, such as living arrangements and travel. Safety concerns (e.g., “safe,” “quarantine,” “precaution”) and modes of travel were more common here than in the ‘Social Participation’ topic. ‘Children and Parenting’ mostly pertained to younger children. “Eligible” was among the most frequent words, reflecting anticipation of vaccines for this demographic. Finally, ‘Personal Relationships’ referenced specific relationships (e.g., “daughter,” “husband,” “friend”) and arrangements or plans (e.g., “stay,” “come,” “live”).

### Theme 6: Navigating the COVID-19 infodemic

This theme contained 5 topics related to interacting with COVID-19 information. Many submissions in ‘Verifying Information’ referenced academic or online sources (e.g., “article,” “post,” “study”) and asked the team to fact-check information. Among the top 50 words in this topic, only “ivermectin” referenced a specific news item, in contrast to general references such as “article” or “news.” ‘Feedback’ captured salutations, post requests, and expressions of gratitude. ‘Data and Statistics’ referenced metrics of COVID-19 burden (e.g., “case,” “death,” “infection”) and reflected confusion about interpreting them. ‘Sense-making’ captured uncertainty and reflected the desire to cope with the pandemic (e.g., “normal,” “want,” “life”). Of note, the word “pandemic” (but not “covid” or “coronavirus”) was among the top 50 words. Finally, ‘Risk Assessment’ captured complex risk decisions (e.g., “understand,” “factor,” “consider”) and reflected desires to simplify them (e.g., “high,” “low,” “reduce”).

### Visualization of question box submissions

**Fig 1:**
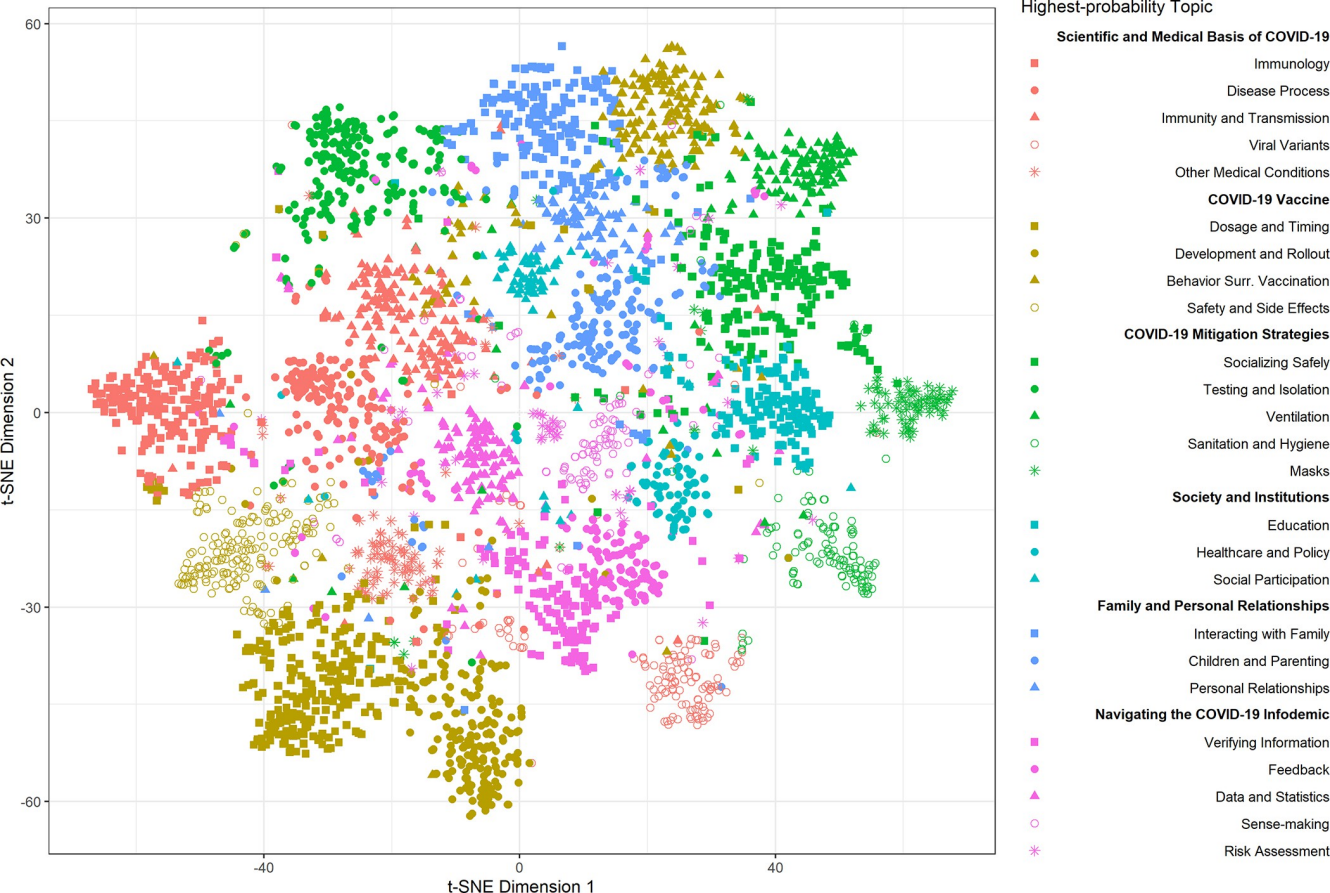
T-SNE visualization of submissions to the Dear Pandemic question box.

Fig 1 displays the t-SNE visualization of submissions and topics. Data points (i.e., submissions) are differentiated by colors and symbols according to their theme and highest-probability topic. Points that are closer in proximity represent submissions with relatively similar topic probability distributions. Overall, the classification of submissions according to their highest-probability topic resulted in well-defined t-SNE clusters. The algorithmic clustering of topics was generally consistent with the themes that we generated using thematic analysis, with three notable exceptions: ‘Testing and Isolation,’ ‘Behavior Surrounding Vaccination,’ and ‘Viral Variants.’ For example, we placed the topic ‘Viral Variants’ under the ‘Scientific and Medical Basis of COVID-19’ theme, but the algorithm clustered submissions in this topic alongside topics in ‘COVID-19 Mitigation Strategies’ and ‘Navigating the COVID-19 Infodemic.’ This demonstrates the interconnectedness among topics and reflects that different thematic classifications may be possible.

### Trend analysis of topic prevalence

**Fig 2:**
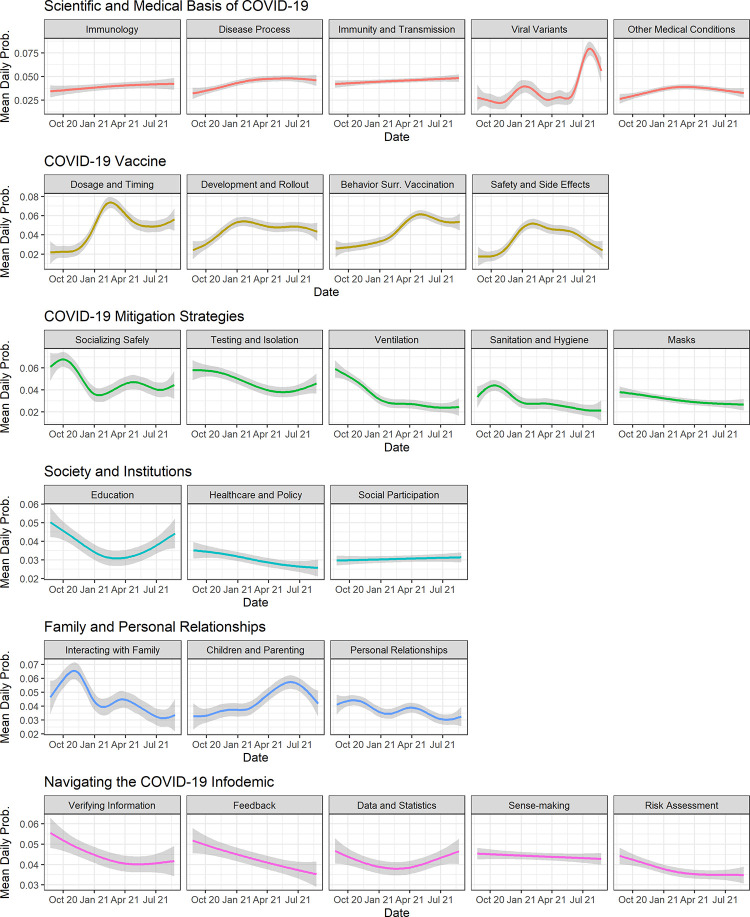
Trends in topic prevalence from August 24, 2020 to August 24, 2021.

Fig 2 displays each topic’s prevalence over time, as determined by its mean daily probability. A number of topics showed distinct trends. ‘Viral Variants’ peaked around January and July 2021, corresponding to increased news attention on the Alpha and Delta variants. Topics in the ‘COVID-19 Vaccine’ theme showed a sequential increase in prevalence over time: first ‘Development and Rollout,’ then ‘Safety and Side Effects,’ then ‘Dosage and Timing,’ then finally ‘Behavior Surrounding Vaccination.’ Conversely, topics in the ‘COVID-19 Mitigation Strategies’ theme decreased in prevalence over time. Finally, topics related to family and children, such as ‘Education,’ ‘Interacting with Family,’ ‘Personal Relationships,’ and ‘Children and Parenting’ peaked around the winter holiday and summer seasons.

## Discussion

In this study, we analyzed questions that readers submitted to Dear Pandemic, a prominent science communication campaign, during the height of the COVID-19 pandemic. We used LDA topic modeling to discover latent topics among the submissions, and we used thematic analysis to interpret the results. We generated six themes that characterized readers’ information needs: ‘Scientific and Medical Basis of COVID-19,’ ‘COVID-19 Vaccine,’ ‘COVID-19 Mitigation Strategies,’ ‘Society and Institutions,’ ‘Family and Personal Relationships,’ and ‘Navigating the COVID-19 Infodemic.’

The topics and themes we identified highlight that Dear Pandemic’s readers sought information not only to explain novel scientific concepts, but also to understand how they applied to their personal lives. For example, the topic ‘Immunology’ included technical words like “antibody,” “autoimmune,” and “cell,” but the questions themselves revealed pragmatic concerns (e.g., “I have 2 autoimmune illnesses, celiac disease & Hashimoto’s thyroiditis. Does that mean I am immunocompromised?”). In their analysis of COVID-19 information seeking on Naver Knowledge iN, Kim & Oh similarly identified topics related to the “cognitive context” (e.g., symptoms and masks) as well as the “situational context” (e.g., financial support, study, and work) [13]. Compared to their study, as well as other studies of general social media discourse [8, 9], we found a higher prevalence of technical terms among the top words (Table 1). This could suggest that the scientific literacy of Dear Pandemic’s readership was higher, or that readers used more technical language to engage with expert science communicators than they would use to engage with the lay public.

Our t-SNE visualization (Fig 1) also suggests ways in which topics were interconnected. As illustrated by the difference between our thematic categorization of certain topics (‘Behavior Surrounding Vaccination,’ ‘Testing and Isolation,’ and ‘Viral Variants’) and the clustering provided by the t-SNE algorithm, quantitative methods can reveal novel insights. For example, submissions related to viral variants were more similar to those surrounding mitigation strategies and the infodemic, rather than the scientific basis of COVID-19. Readers may have been more immediately concerned with, for instance, assessing and mitigating risks due to the Delta variant than with understanding the science behind its emergence. This reinforces the need for science communicators to offer practical, timely guidance–not just science lessons. In their study of psychosocial stressors during the pandemic, Leung & Khalvati similarly used t-SNE to visualize the association between latent topics in Reddit posts [23]. They found that family-related topics clustered with topics about the fear of COVID-19. In our analysis, family-related topics clustered with topics about personal behavior (e.g., ‘Behavior Surrounding Vaccination,’ ‘Socializing Safely,’ and ‘Testing and Isolation’). Based on these findings, science communicators may hypothesize that family-related worries about COVID-19 prompt online audiences to seek information about how their family members can modify their behavior, and tailor their content accordingly.

The prevalence of topics related to family and children in our results is unsurprising in light of Dear Pandemic’s modal readers: women aged 35–54 years old. Women generally seek online health information more often than men [24]. In an analysis of COVID-19 related worries, van der Vegt & Kleinberg found that women disproportionately worried about family members and severe health consequences, whereas men disproportionately worried about the economy and society [25]. Interestingly, economic concerns did not emerge as a distinct topic in our analysis, which may reflect this demographic trend. In contrast to other analyses conducted using Facebook posts or Tweets [9, 26], we also did not identify any topics related to politics, pseudoscience, or conspiracy theories. This could be due to differences in audience characteristics (e.g., scientific literacy, partisanship, or preferred information sources). It is important to counter misinformation through engagement with skeptics, but it is also critical to recognize that even those who are highly engaged with trusted science communicators and attuned to evidence-based guidance have unmet information needs.

As revealed in our trend analysis (Fig 2), Dear Pandemic readers sought information about certain issues, such as the emergence of viral variants, in line with news media attention. Readers also sought information in anticipation of upcoming events in their lives, as indicated by peaks in topics related to children or family prior to the start of the school year or the winter holidays. Finally, the sequential rise of topics within the ‘COVID-19 Vaccine’ theme–from ‘Development and Rollout,’ to ‘Safety and Side Effects,’ to ‘Dosage and Timing,’ to ‘Behavior Surrounding Vaccination’–mirrored the real-world progression of vaccine-related events. Kim & Oh similarly found that COVID-19 information needs related to the “social context” rose in prevalence later than information needs related to the “cognitive” and “situational” contexts [13]. Science communicators should thus be mindful of these temporal factors when fulfilling information needs.

Our results offer significant contributions to the literature on online COVID-19 discourse. To the best of our knowledge, we are the first to analyze text written for the specific purpose of seeking information from trusted experts. Most other studies used pre-determined hashtags to extract COVID-19 discourse from platforms such as Twitter [7, 9, 10], or used search trends as a proxy for information needs [11]. These studies were able to capture a wide breadth of discourse, but they lacked the ability to discern the purpose for which it was written. For example, Abd-Alrazaq et al. identified topics such as deaths caused by COVID-19, economic losses, and mask usage in a seminal infodemic surveillance study of Twitter discourse [7]. While they were able to compute prevalence statistics, sentiment scores, and engagement metrics for each topic, we note that their use of a non-specific dataset meant that their results may not have necessarily reflected information needs. Science communicators may therefore have difficulty translating the results of such studies into practice. In contrast, Kim & Oh were able to more plausibly conclude that the topics they identified among posts to the question-and-answer platform Naver Knowledge iN reflected users’ information needs [13]. Since our study explicitly focused on readers who contacted science communicators about their information needs, our insights may be even more readily translated into practice.

More broadly, we demonstrate that Dear Pandemic’s question box format and our analytical approach can be adopted by other science communicators and researchers. These tools align with the WHO’s infodemic management framework, which recommends mixed-methods protocols for analyzing information flows [5]. The question box format, which facilitates two-way engagement with readers, generates rich text data that can be used in LDA topic models. One significant advantage of using such quantitative methods to analyze the data is that they are more time- and resource-efficient. If researchers want to glean deeper insights, however, we show that they can certainly integrate topic modeling with qualitative methods such as thematic analysis. Our longitudinal analysis also shows that topic modeling can be useful in real time, as we found that trends in topic prevalence co-occurred with developments in the COVID-19 information landscape. We note that dynamic topic models may be better-suited to identify real-time information needs in practice, as they can quickly track the evolution of individual topics, but these methods require larger datasets than we used for our study [8].

We also acknowledge our study’s limitations. Regarding our data, limiting our corpus to English-language webform submissions excluded attempts that readers made to seek information in other languages and through other channels. In order to protect anonymity and promote trust from readers, the webform did not request any demographic information beyond location. Information-seeking habits on social media are associated with demographic and personality characteristics [27], so Dear Pandemic readers who submitted questions likely differed from those who did not. Regarding our methods, our decision to use a 25-topic model was partially based on our subjective evaluation of semantic validity and granularity, in addition to C_v_ score. A 9-topic model would have yielded the highest C_v_ score, but we judged the topics to be insufficiently granular; selecting a different number of topics may have yielded different conclusions. Though we subsequently followed an established process of thematic analysis to interpret the results of our LDA topic model, it has known limitations, including its inherently subjective nature and inability to allow for much interpretation beyond description [17, 22]. Finally, we recognize that the social media landscape is fragmented and heterogeneous, so our findings may not be generalizable to other platforms.

## Conclusions

In our continued efforts to manage the COVID-19 pandemic, we should also strive to manage the parallel infodemic. Our analysis of submissions to the Dear Pandemic question box adds to the literature on online COVID-19 discourse, focusing on the information needs of online audiences who engage with trusted science communicators. Our topic model and thematic analysis demonstrates that such audiences seek information that is scientifically rigorous, timely, and practical to their daily decisions and personal circumstances. This mixed-methods approach offers a model for other science communicators who aim to establish a mechanism for tracking, understanding, and responding to their readers’ information needs. Future studies should continue to characterize the information needs of different online audiences during the COVID-19 pandemic, as well as the ways in which science communicators and other entities have attempted to fulfill them.

## Supporting information

S1 File R analysis code. (R)

S1 Data Geographic location of Dear Pandemic question box submissions. (CSV)

S2 Data Full topic probability distribution for Dear Pandemic question box submissions. (CSV)

S3 Data Topics, themes, and top words. (CSV)

S1 Fig Overview of research process. (TIF)

S2 Fig C_v_ coherence scores for LDA models with 3 to 50 topics. (TIFF)

S3 Fig Daily submissions to the Dear Pandemic question box, August 24, 2020 to August 24, 2021. (TIFF)
