# Supplementary figures and images for: Dear Pandemic: A topic modeling analysis of COVID-19 information needs among readers of an online science communication campaign
Source: PLoS One. 2023 Mar 30;18(3):e0281773. doi: 10.1371/journal.pone.0281773 (PMC10062627; doi:10.1371/journal.pone.0281773)

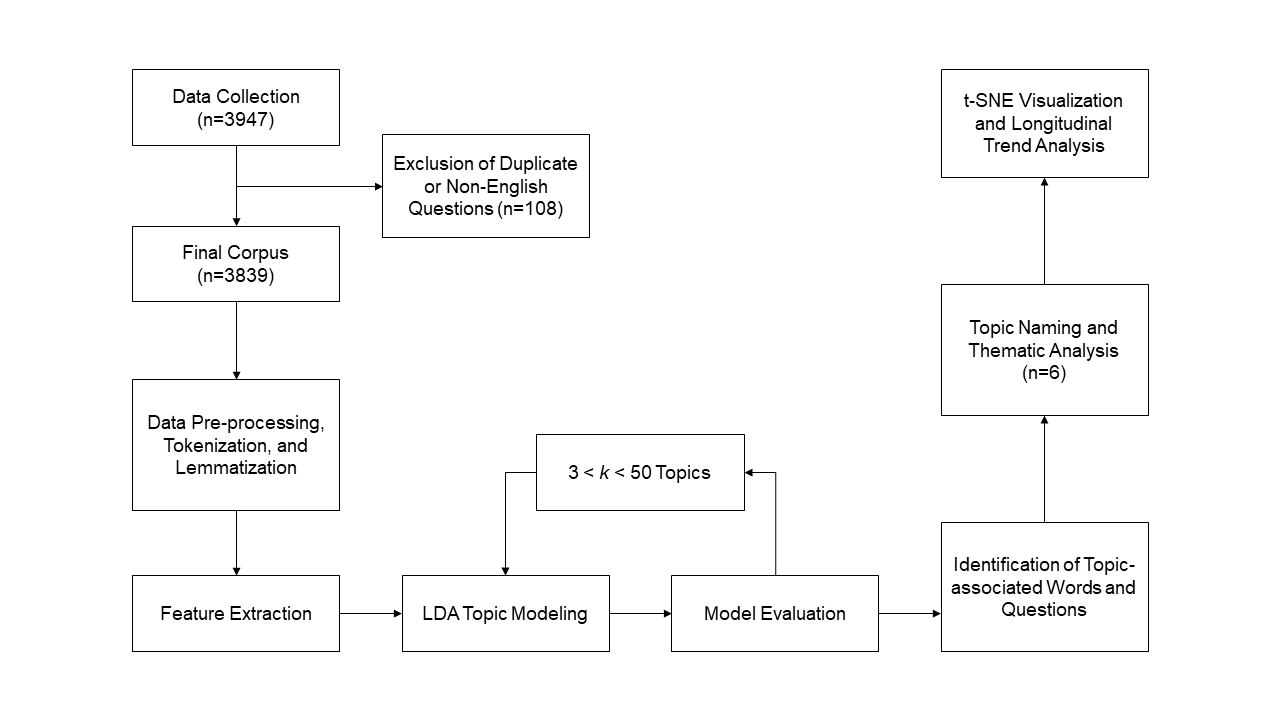

Supplement: S1 Fig — (TIF) [file pone.0281773.s005.tif]

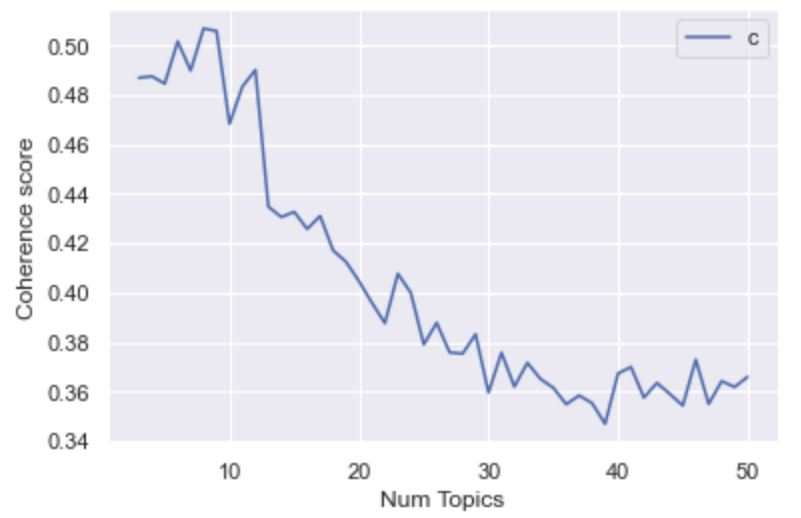

Supplement: S2 Fig — (TIFF) [file pone.0281773.s006.tiff]

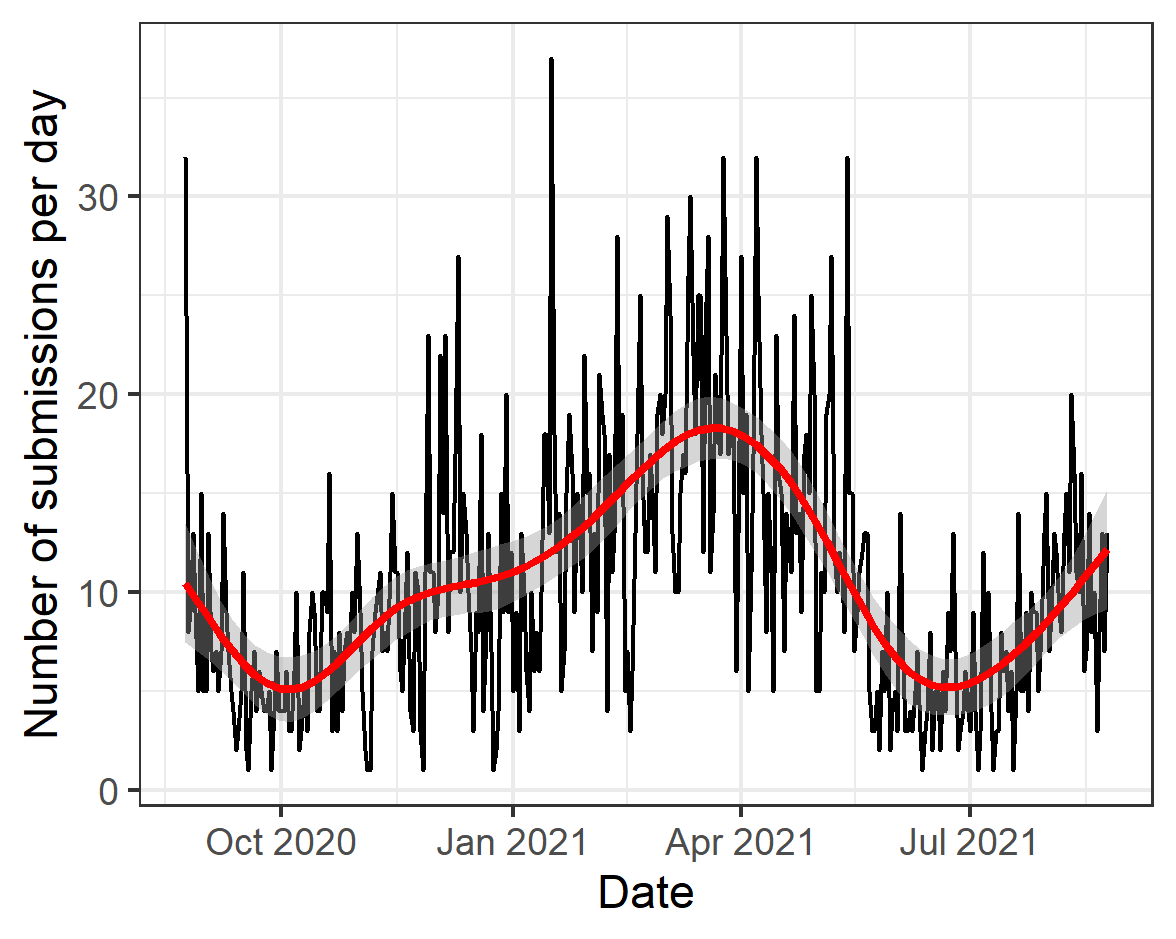

Supplement: S3 Fig — (TIFF) [file pone.0281773.s007.tiff]
